# Biochemical Analysis of Histone Succinylation

**DOI:** 10.1155/2017/8529404

**Published:** 2017-11-01

**Authors:** Atsushi Yokoyama, Shogo Katsura, Akira Sugawara

**Affiliations:** ^1^Department of Molecular Endocrinology, Tohoku University Graduate School of Medicine, 2-1 Seiryo-machi, Aoba-ku, Sendai 980-8575, Japan; ^2^Institute of Molecular and Cellular Biosciences, University of Tokyo, Yayoi, Bunkyo-ku, Tokyo 113-0032, Japan

## Abstract

Posttranslational modification (PTM) of proteins is used to regulate protein activity and stability. Histone PTMs are regarded as some of the most important, as they can directly regulate gene expression through chromatin reorganization. Recently, histone proteins were found to undergo succinylation, adding to other well-known PTMs such as acetylation, methylation, and phosphorylation. However, there is little information regarding the enzyme which catalyzes histone lysine succinylation. In fact, it is unclear whether this reaction is enzymatic. In this study, we tested histone succinylation activity* in vitro* using cell nuclear extracts of HepG2 cells. Although whole nuclear extracts did not show histone succinylation activity, we found that an SP 1.0 M KCl fraction of nuclear extracts indeed had such activity. These data offer the first direct evidence that histone succinylation is an enzymatic PTM as are other histone codes in the nucleus.

## 1. Introduction

Since the first identification of *ε*-acetylation of lysine in histone proteins [1, 2], several hundred protein posttranslational modifications (PTMs) have been identified in both histone and nonhistone proteins. These include methylation, phosphorylation, glycosylation, and acylation [3, 4]. The role of PTMs in protein transportation, DNA repair, and gene regulation is apparent. Among them, PTMs of histone proteins are regarded as extremely important because they can directly regulate gene expression through chromatin reorganization [5–7].

Histone proteins are dynamically modified at specific amino acid residues by a variety of histone-modifying enzymes such as histone acetyl transferases (HATs) and histone methyl transferases (HMTs). These modifying groups are removed by their cognate enzymes (e.g., histone deacetylases (HDACs) and histone demethylases (HDMs)) [6, 8]. Adding to well-characterized PTMs (acetylation, methylation, phosphorylation, and ubiquitination), several types of histone acylation have been reported, such as propionylation, butyrylation, crotonylation, 2-hydroxyisobutyrylation, glutarylation, malonylation, and succinylation [9–11]. A linkage between histone acylation and transcriptional activation has been shown [12–14], although the function of histone acylation in chromatin reorganization remains elusive.

To catalyze protein modification, many histone-modifying enzymes utilize coenzymes that are derived from cellular metabolic reactions, providing a link between the cellular metabolic state and gene regulation [15]. For example, for histone lysine acetylation, HATs (such as p300 and CBP) transfer acetyl groups from acetyl-CoA, a key metabolic coenzyme, to the *ε*-amino group of the lysine residue. On the other hand, sirtuin 1 (SIRT1), one of the HDACs, removes the acetyl group from histone lysine residues using NAD^+^ as a cofactor [15]. Recently, histone lysine succinylation, an acylation reaction, was discovered using a mass spectrometric approach [16]. Although SIRT5 was identified as a responsible “eraser” for lysine succinylation in mitochondria [17, 18], the enzymes that regulate histone lysine succinylation in the nucleus remain elusive. Furthermore, some studies claim that lysine succinylation in mitochondria is not an enzymatic PTM [19, 20]. Therefore, there is little information regarding the enzyme that catalyzes histone lysine succinylation or even whether this reaction is in fact enzyme-dependent.

In this study, we tested histone succinylation activity* in vitro* using nuclear extracts. Although whole nuclear extracts did not show histone succinylation activity, we found that the strong cation exchange column-binding fraction (HiTrap SP column, 1.0 M KCl-eluted fraction) of nuclear extracts indeed possessed succinylation activity. These data could provide direct evidence that histone succinylation requires an enzymatic PTM as do other histone codes in the nucleus.

## 2. Materials and Methods

### 2.1. Reagents

^14^C-labeled acyl-coenzyme A (acetyl-CoA, malonyl-CoA, and succinyl-CoA) was purchased from PerkinElmer (Waltham, MA, USA). Calf thymus histone and unlabeled acyl-coenzyme A were purchased from Sigma-Aldrich (St. Louis, MO, USA). For western blotting, the following antibodies were used: anti-IKK*α* (Santa Cruz, sc-7218) (CA, USA), anti-HDAC2 (ABR, PA I-861) (Golden, CO, USA), anti-acetyl histone H3 (Millipore, 06-594) (Bedford, MA, USA), anti-E1A-binding protein p300 (p300) (Santa Cruz, sc-585), and anti-CREB-binding protein (CBP) (Abcam, ab3652) (Cambridge, UK).

### 2.2. Cell Culture

HepG2 (ATCC number HB-8065) cells were obtained from the ATCC (Baltimore, MD, USA) and cultured in DMEM (Wako, Osaka, Japan) supplemented with 10% fetal bovine serum and antibiotics (100 units/mL penicillin G and 100 *μ*g/mL streptomycin, Wako). The cells were grown at 37°C in 5% CO_2_ as previously described [21] and harvested after 1 day of incubation from the last passage.

### 2.3. Western Blotting

Protein extracts, separated by SDS-PAGE and transferred onto PVDF membranes (Millipore), were probed with antibodies against indicated proteins. Proteins of interest were detected with HRP-conjugated donkey anti-rabbit IgG antibody (GE Healthcare, Uppsala, Sweden) and developed with the Clarity Western ECL Substrate (Bio-Rad, Hercules, CA, USA) and BioMax XAR film (Kodak, Rochester, NY, USA).

### 2.4. Cell Fractionation

Each cell fraction was prepared as previously described [22–24]. Briefly, HepG2 cells in 24  10-cm dishes were collected with a cell scraper. Cells were washed with ice-cold PBS and then with hypotonic buffer (10 mM HEPES, pH 7.6, 1.5 mM MgCl_2_, and 10 mM KCl). The washed cell pellets were incubated with 3 CPV (packed cell volume) of hypotonic buffer on ice for 10 min. Swollen cells were homogenized with a Dounce homogenizer (Wheaton, Millville, NJ, USA) with a loose pestle and centrifuged at 3,300 ×g for 15 min. The supernatant was dialyzed overnight against BC100 buffer (20 mM HEPES, pH 7.6, 10% glycerol, 0.2 mM EDTA, and 100 mM KCl) and retained as a cytosolic extract at −80°C. Pelleted nuclei were resuspended in a volume of low-salt buffer (20 mM HEPES, pH 7.6, 10% glycerol, 1.5 mM MgCl_2_, 0.2 mM EDTA, and 20 mM KCl) equal to 1/2 PNV (packed nuclear volume). A volume of high-salt buffer (20 mM HEPES, pH 7.6, 10% glycerol, 1.5 mM MgCl_2_, 0.2 mM EDTA, and 1.2 M KCl) equal to 1/2 PNV was then added to the solution in a dropwise fashion, while stirring gently for 30 min. Samples were centrifuged at 25,000 ×g for 30 min, and the supernatants were dialyzed overnight against BC100 buffer and stored at −80°C as nuclear extracts. Finally, pellets were washed with ice-cold PBS and resuspended in chromatin buffer (20 mM HEPES, pH 7.6, 0.3 M Sucrose, 2 mM MgCl_2_, 1 mM CaCl_2_, 100 mM KCl, and 0.1% Triton X-100). Micrococcal nuclease (Takara Bio, Shiga, Japan) was added to a final concentration of 30 U/mL and incubated for 30 min at room temperature with occasional homogenization. The reaction was stopped by adding EDTA to a final concentration of 5 mM, and fractions were stored at –80°C as chromatin extracts. For ion exchange column separation, nuclear extracts were applied to a HiTrap SP column (GE Healthcare) with the AKTA purifier system (GE Healthcare) and eluted by stepwise elution (KCl: 0.1, 0.3, 0.6, and 1.0 M). Each eluted fraction was dialyzed overnight against BC100 buffer.

### 2.5. *In Vitro* Acylation Reaction

*In vitro* histone acylation was assessed by detecting acyl-CoA incorporation described by Renstrom and Deluca in 1989 [25] with some modifications. Total reaction mixtures (250 *μ*L) contained the following: 30 mM Tris, pH 7.5, 50 nCi ^14^C-labeled acyl-coenzyme A, 1 mM  2-mercaptoethanol, and 300 *μ*g of a protein extract with or without 2 *μ*g of calf thymus histone. Protein concentration was measured with Protein Assay (Bio-Rad) using bovine serum albumin (Roche Diagnostics, Indianapolis, IN, USA) as a standard. After incubation at 37°C for 30 min, the proteins were extracted by methanol/chloroform precipitation. Precipitated proteins were dried and resuspended in 2% SDS. Radioactivities of the samples were assessed with a liquid scintillation counter (Beckman Coulter, Brea, CA, USA) or autoradiography.

## 3. Results and Discussion

**Figure 1:**
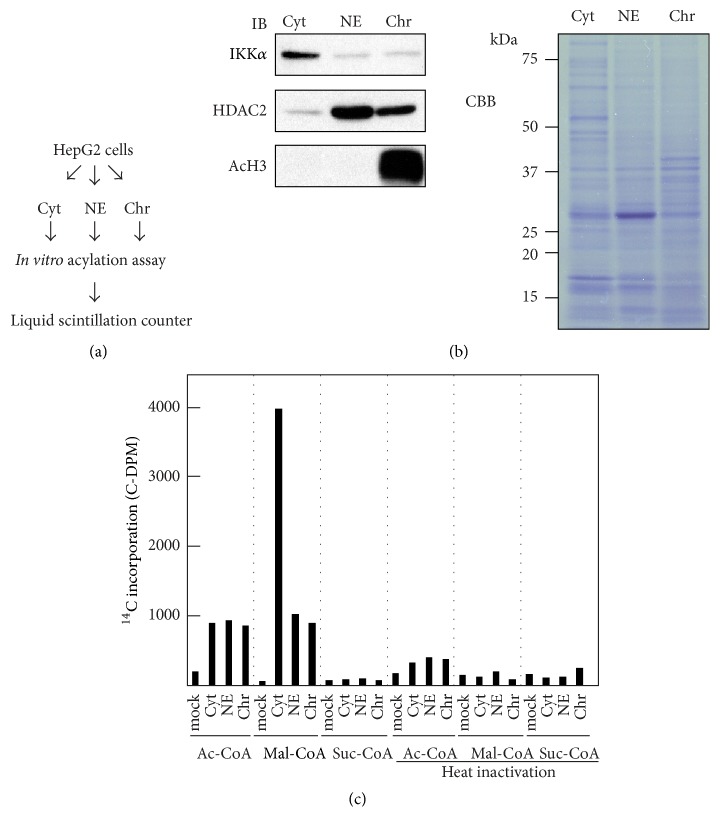
*In vitro* acylation assay using fractionated HepG2 cell extracts. (a) Schematic diagram of the fractionation of HepG2 cell extracts and* in vitro* acylation assay. (b) Validation of HepG2 cells fractionated into cytosolic, nuclear, and chromatin extracts. 4 *μ*g of each extract was assessed by western blot (left panel). IKK*α*, HDAC2, and AcH3 were used as cytosolic, nuclear, and chromatin fraction markers, respectively. CBB staining of loading proteins is shown in the right panel. Cyt: cytosolic extracts; NEs: nuclear extracts; Chr: chromatin extracts. (c)* In vitro* acylation assays using fractionated cell extracts. ^14^C-labeled coenzyme A incorporation was assessed by liquid scintillation counting. Reaction mixtures without cell extracts were used as negative controls (mock). To show that the incorporation was an enzymatic reaction, cell extracts were heat-inactivated at 96°C for 10 min. Ac-CoA: acetyl-coenzyme A; Mal-CoA: malonyl-coenzyme A; Suc-CoA: succinyl-coenzyme A.

To test cellular succinylation activity* in vitro*, we first separated HepG2 cells into three fractions: cytosolic, nuclear, and chromatin extracts (Figure 1(a)). As shown in Figure 1(b), all extracts were successfully fractionated, as each compartment was enriched for the indicated marker. To detect protein succinylation activity, we initially incubated the 3 HepG2 cell fractions with ^14^C-labeled acyl-coenzyme A at 30°C. After the reaction was stopped, proteins were extracted from the reaction mixture by methanol-chloroform precipitation and those extracted proteins were analyzed for incorporation of labeled acyl-coenzyme A as assessed by liquid scintillation activity. In this experiment, we utilized acetyl-coenzyme A (Ac-CoA) as a positive control for acylation reaction. As for Ac-CoA, all three fractions showed Ac-CoA incorporation into proteins compared to the control. Heat inactivation of reaction mixtures before incubation inhibited incorporation. This result suggested that the extracts contained enzymes and substrates for protein acetylation and that the reaction was enzymatic as it is well known. Under these conditions, we tested the incorporation of malonyl-coenzyme A (Mal-CoA) and succinyl-coenzyme A (Suc-CoA). As shown in Figure 1(c), all three fractions, especially the cytosolic extracts, exhibited robust Mal-CoA incorporation. Heat inactivation inhibited this incorporation, suggesting that this reaction was enzymatic, as was acetylation. On the other hand, Suc-CoA did not show incorporation in the extracts as did Ac-CoA and Mal-CoA.

**Figure 2:**
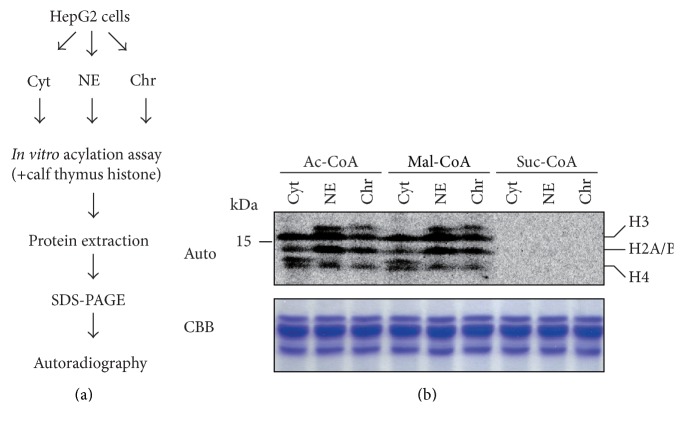
*In vitro* histone acylation assay using fractionated HepG2 cell extracts. (a) Schematic diagram of the fractionation of HepG2 cell extracts and* in vitro* acylation assay using calf thymus histone. (b)* In vitro* histone acylation assays using fractionated cell extracts. ^14^C-labeled coenzyme A incorporation into histone proteins was assessed by autoradiography. The amounts of added substrate (calf thymus histone proteins) in each reaction were visualized by Coomassie Brilliant Blue (CBB). Molecular weights are indicated in the left side of the figure, and each histone position (histones H3, H2A/B, and H4) is indicated on the right side of the figure.

Next, we tested whether these fractions had histone acylation activity. Calf thymus histones were incubated with each extract in the presence of ^14^C-labeled acyl-coenzyme A at 30°C for 30 min. After the reaction was stopped, all proteins were precipitated and washed to remove unreacted labeled compounds. Precipitated proteins were then subjected to SDS-PAGE and autoradiography to detect acyl-CoA incorporation (Figure 2(a)). As in the results of liquid scintillation counter analysis in Figure 1(c), Ac-CoA and Mal-CoA were strongly incorporated into all histone proteins (Figure 2(b)). However, Suc-CoA did not show incorporation into the histone proteins (Figure 2(b)). The latter results could be due to one or more of the following: (1) protein succinylation might not be enzyme-based, (2) the extracts contained factors inhibiting protein succinylation, or (3) desuccinylation activity might be dominant in these extracts.

**Figure 3:**
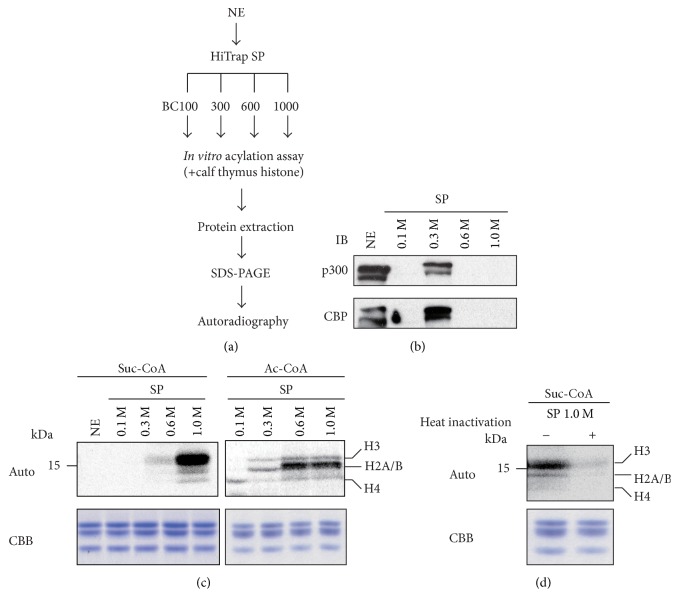
*In vitro* histone acylation assays using strong cation exchange column-fractionated HepG2 nuclear extracts. (a) Schematic diagram of the fractionation of HepG2 nuclear extracts (NEs). HepG2 NEs were loaded on the HiTrap SP column and eluted with BC buffers containing the indicated KCl concentration. Each eluted protein was used for the* in vitro* acylation reaction. (b) Validation of HepG2 cell nuclear extracts fractionation by strong cation exchange column. Nuclear extract (NE) input and each eluted fraction (0.1, 0.3, 0.6, and 1.0 M KCl) were assessed by western blotting using anti-p300 and anti-CBP as first antibodies. (c) ^14^C-labeled coenzyme A incorporation into histone proteins was assessed by autoradiography. The amounts of added substrate (calf thymus histone proteins) in each reaction were visualized by Coomassie Brilliant Blue (CBB). Molecular weight is indicated on the left side of the figure, and each histone position (histones H3, H2A/B, and H4) is indicated in the right side of the figure. (d) The effect of heat inactivation was assessed. The SP 1.0 M fraction of nuclear extracts was heat-inactivated at 96°C for 10 min. The amounts of added substrate (calf thymus histone proteins) in each reaction were visualized by Coomassie Brilliant Blue (CBB). Molecular weight is indicated on the left side of the figure, and each histone position (histones H3, H2A/B, and H4) is indicated in the right side of the figure. SP: HiTrap SP column.

To assess whether histone succinylation was indeed enzymatic, we further fractionated the nuclear extracts into four fractions depending on their binding property to a strong cation exchange column (Figure 3(a)). In this fractionation, p300 and CBP, well-known histone acetyl transferases, were eluted exclusively at 0.3 M KCl (Figure 3(b)), indicating successful nuclear extract fractionation. Next, we utilized these fractions for the* in vitro* histone modification assay. The input nuclear extracts and the fractions eluted at 0.1, 0.3, and 0.6 M KCl did not have histone succinylation activities in contrast to the histone acetylation activity (Figure 3(c)). However, surprisingly, the fraction eluted with 1.0 M KCl possessed histone succinylation activity especially for histone H3 (Figure 3(c)), and heat inactivation inhibited this incorporation (Figure 3(d)). This result implies that this fraction from the HepG2 nuclear extracts contains one or more enzymes other than p300 and CBP that possessed histone succinylation activity.

**Figure 4:**
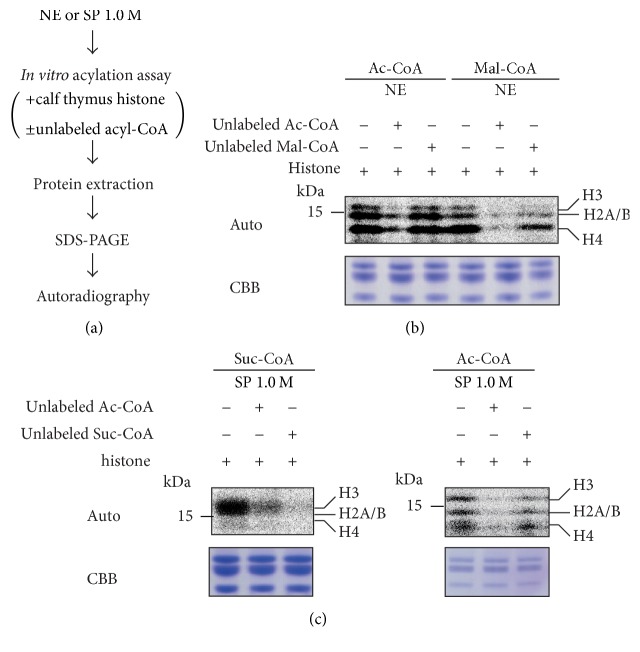
Competition assay for acyl-coenzyme A incorporation into histone proteins. (a) Schematic diagram of* in vitro* acylation assay with unlabeled acyl-CoA for substrate competition.* In vitro* histone acylation assays with nonlabeled acyl-coenzyme A. ^14^C-labeled coenzyme A incorporation into histone proteins in the presence of an excess amount (5 nmol) of nonlabeled malonyl-coenzyme A (b) and succinyl-coenzyme A (c) was assessed by autoradiography. Acetyl-coenzyme A was used as a control for the substrate specificity. The amounts of added substrate (calf thymus histone proteins) in each reaction were visualized by Coomassie Brilliant Blue (CBB). Molecular weights are indicated on the left side of the figure, and each histone position (histones H3, H2A/B, and H4) is indicated in the right side of the figure.

In these* in vitro* assays, there was a concern that ^14^C-labeled acyl-CoA might be metabolized to a related molecule such as Ac-CoA in the extracts and then incorporated into the histone proteins as acetylation. To confirm that these* in vitro* histone modification assays indeed detected the expected incorporation of labeled acyl-CoA, we utilized an excess amount of unlabeled acyl-CoA (unlabeled acyl-CoA) for substrate competition (Figure 4(a)). In Figure 4(b), Ac-CoA was clearly outcompeted by unlabeled Ac-CoA but not by unlabeled Mal-CoA, indicating specific histone acetylation. On the other hand, histone malonylation was also completely outcompeted by Ac-CoA as well as Mal-CoA. This result suggests that the reaction using Mal-CoA might detect histone acetylation. In Figure 4(c), left panel, the histone succinylation reaction using the SP 1.0 M fraction was completely outcompeted by unlabeled Suc-CoA. Similarly, the histone acetylation activity of the fraction was outcompeted specifically by unlabeled Ac-CoA (Figure 4(c), right panel). Although unlabeled Ac-CoA also showed some competition for Suc-CoA incorporation, this was not the complete competition that was seen in Mal-CoA incorporation, suggesting that Suc-CoA was incorporated into histone protein.

After discovery of protein lysine succinylation [26], many mitochondrial proteins were reported to be succinylated and functionally regulated in this way [27]. SIRT5 was identified as the protein responsible for removal of succinyl substitutions in mitochondria [17, 18]. The enzyme that catalyzes histone succinylation in mitochondria has yet to be identified. On the other hand, in the presence of high concentrations of acyl-CoA and elevated pH, the possibility of nonenzymatic lysine succinylation in mitochondria has been proposed [19, 20]. However, information about the histone succinylation reaction in the nucleus remains scarce. That is, the enzymes responsible for histone succinylation have not been identified, and it is not clear whether histone succinylation requires enzymatic catalysis. In this report, we have demonstrated for the first time that a fraction of the HepG2 nuclear extract has histone succinylation activity* in vitro*.

Here, we found that total nuclear extracts did not possess histone succinylation activity. We assume that this is because the total nuclear extract contained inhibitory factor(s) for the reaction or that desuccinylation activity might be dominant in these extracts. Though a histone desuccinylase enzyme has not been identified, given that mitochondrial SIRT5 has lysine desuccinylation activity in mitochondria, lysine deacetylases such as nuclear localized HDAC or SIRT family proteins are possible histone desuccinylases [17]. The identification of decrotonylation activity of Class I HDACs also supports the hypothesis that a histone deacetylase family protein could remove acyl moieties [28, 29].

In Figure 3(b), p300 and CBP were detected in the fraction eluted by 0.3 M KCl, but it did not show succinylation activity. However, there are reports that p300 has histone succinylation activity* in vitro *[17, 30, 31]. In terms of succinylation activity, our results do not agree with those earlier reports. This discrepancy is presumably due to differences in experimental conditions, especially with regard to the substrates used. Concerning the substrate types, we used calf thymus histones as histone succinylation substrates, whereas others used synthetic peptides harboring a histone tail sequence. The responsible enzyme activity in the nuclear fraction eluted with 1.0 M KCl remains elusive and the identification of this factor will be pursued further.
